# Synthesis and structure of 2-amino-4-nitro-1*H*-imidazol-3-ium chloride

**DOI:** 10.1107/S2056989025011399

**Published:** 2025-01-23

**Authors:** Jun Yuan, Liping Lin, Xinzhi Wang, Ling Qin, Xiao Li, Guanchao Lan, Lizhen Chen, Jianlong Wang

**Affiliations:** a School of Chemistry and Chemical Engineering North University of China,Taiyuan 030051 People’s Republic of China; b Gansu Yin Guang Chemical Industry Group Co Ltd Baiyin 730900 People’s Republic of China; Universidade Federal do ABC, Brazil

**Keywords:** crystal structure, 2-amino-4-nitro­imidazole hydro­chloride, hydrogen bonding, supra­molecular architecture, Hirshfeld surface analysis

## Abstract

Single crystals of 2-amino-4-nitro-1*H*-imidazol-3-ium chloride, C_3_H_5_N_4_O_2_^+^·Cl^−^, were synthesized by slow evaporation and characterized by X-ray diffraction. The crystal structure features a planar imidazolium cation and a chloride anion, forming a three-dimensional supra­molecular network *via* N—H⋯Cl and N—H⋯O hydrogen bonds. Hirshfeld surface analysis reveals that O⋯H/H⋯O contacts (28.7%) and Cl⋯H/H⋯Cl inter­actions (24.2%) dominate the inter­molecular packing, with C⋯O/O⋯C contacts (5.1%) indicating significant C—H⋯O hydrogen-bonding contributions. Additional consolidation arises from van der Waals inter­actions (H⋯H, 13.4%), while π–π stacking remains negligible. The synergy of strong hydrogen bonds and weaker inter­actions underpins the robust supra­molecular architecture, providing insights into crystal engineering strategies for nitro­imidazole-based functional materials.

## Abstract 2

Single crystals of 2-amino-4-nitro­imidazole hydro­chloride were synthesized by slow evaporation and characterized by X-ray diffraction. The crystal structure features a planar imidazolium cation and a chloride anion, forming a three-dimensional supra­molecular network via N—H⋯Cl and N—H⋯O hydrogen bonds.

## 1. Chemical context

2-Amino-4-nitro­imidazole hydro­chloride represents an important nitro­imidazole derivative whose physicochemical properties are significantly influenced by hydro­chloride salt formation. The incorporation of a chloride counter-ion establishes characteristic charge-assisted hydrogen-bonding networks (Brammer *et al.*, 2001 ▸), a structural motif widely recognized to enhance stability and modify solubility profiles in pharmaceutical salts (Childs *et al.*, 2007 ▸). The structural chemistry of such hydro­chloride salts consistently reveals robust N—H⋯Cl hydrogen-bonding patterns that dictate mol­ecular organization (Aakeroy *et al.*, 2010 ▸), generating extended supra­molecular architectures through well-defined synthons (Desiraju, 1995 ▸).

The presence of complementary nitro and amino substituents creates a complex electronic environment where the nitro group serves as a multifunctional hydrogen-bond acceptor in both strong N—H⋯O and weaker C—H⋯O inter­actions (Etter, 1990 ▸). This versatile hydrogen-bonding capability aligns with established crystal engineering principles (Laus *et al.*, 2006 ▸), where protonation-induced modification of the electron distribution creates distinctive inter­molecular inter­action patterns (Bernstein *et al.*, 1995 ▸). These structural features directly influence thermal stability and decomposition pathways, particularly relevant for energetic materials applications (Bolton *et al.*, 2012 ▸). From a biological perspective, the enhanced aqueous solubility of hydro­chloride salts frequently translates to improved bioavailability, making them preferred forms for pharmaceutical development (Phan *et al.*, 2016 ▸). This structural paradigm demonstrates how targeted manipulation of inter­molecular inter­actions enables precise control over solid-state properties (Cruz-Cabeza *et al.*, 2015 ▸). The present work reports the synthesis and crystal structure of 2-amino-4-nitro­imidazole hydro­chloride.
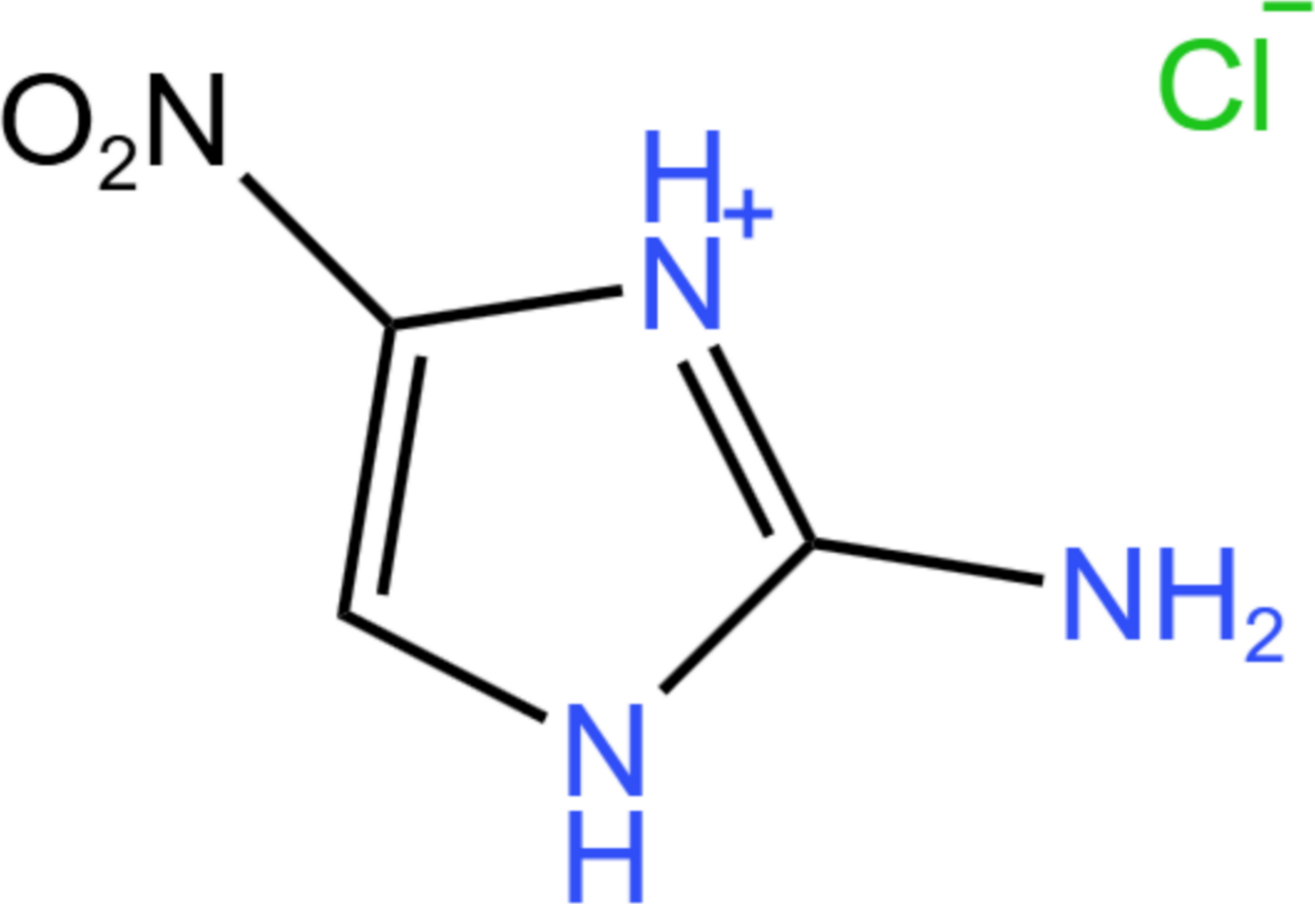


## 2. Structural commentary

**Figure 1:**
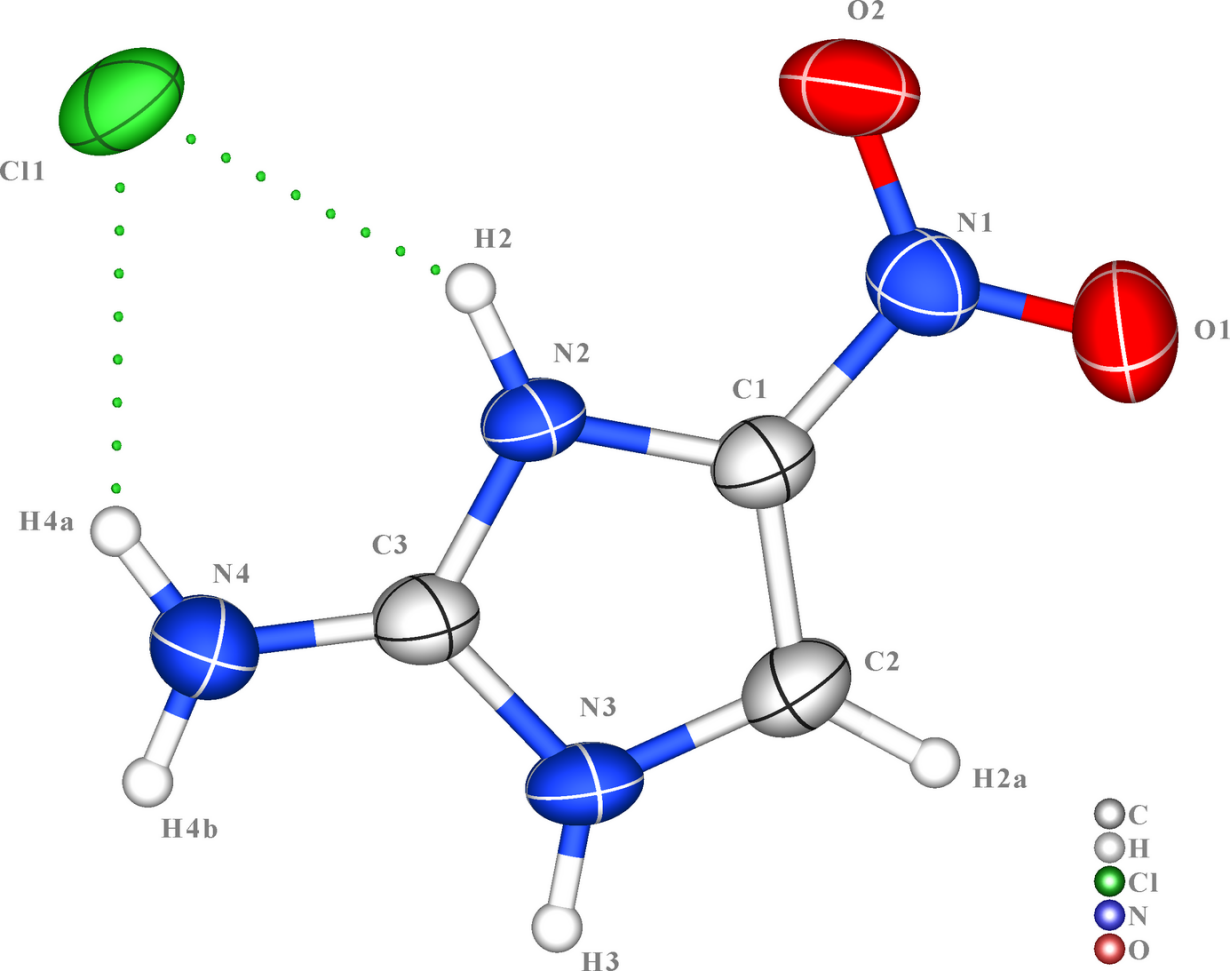
The mol­ecular structure of 2-amino-4-nitro­imidazole hydro­chloride with displacement ellipsoids drawn at the 50% probability level. The dashed line indicates the hydrogen bond forming an *S*(6) pseudo-ring.

As shown in Fig. 1 ▸, the asymmetric unit of the crystal structure consists of one 2-amino-4-nitro­imidazolium cation and one chloride counter-anion. Structural analysis reveals that when the imidazole ring is modified with small substituents such as amino and nitro groups, it exhibits excellent overall planarity. The core imidazolium ring system (comprising atoms N2, C1, C2, N3, C3) shows near-perfect planarity with a root-mean-square (r.m.s.) deviation of merely 0.002 (1) Å from the least-squares plane. Furthermore, the amino group at the C3 position and the nitro group at the C1 position demonstrate high coplanarity with the heterocyclic ring, with dihedral angles of only 0.5 (2) and 2.8 (2)°, respectively. This exceptional flatness, achieved by the amino-nitro substitution pattern, provides an ideal foundation for electronic delocalization and robust inter­molecular inter­actions, which is crucial for forming well-defined supra­molecular architectures. This phenomenon is consistent with previous findings showing that other small substituents (*e.g*., methyl, halogens) generally preserve the ring’s planarity (Li *et al.*, 2008 ▸; Palumbo *et al.*, 2008 ▸; Kannoujia *et al.*, 2023 ▸; Andra *et al.*, 2010 ▸). In stark contrast, bulky groups like adamantyl induce significant ring distortion due to steric hindrance (Cabildo, 1985 ▸).

The C3—N4 bond length of 1.310 (2) Å provides compelling evidence for substantial electron delocalization across the N4—C3—N2 fragment. This value is significantly shorter than a typical C—N single bond (1.47 Å) and approaches the length of a formal C=N double bond, indicating pronounced double-bond character resulting from resonance inter­actions. This phenomenon, well-documented in 2-amino­imidazole derivatives (Tabatabaee *et al.*, 2012 ▸), involves delocalization of the amino nitro­gen lone pair into the aromatic system, generating partial double-bond character between N4 and C3. The near-perfect coplanarity of the amino group, with N4 deviating only 0.008 (3) Å from the ring plane, provides complementary structural evidence for this π delocalization, as optimal orbital overlap for conjugation requires this spatial alignment. The chloride anion resides in close proximity to the cationic plane, positioned 0.124 (3) Å from the mean plane of the imidazolium ring, facilitating strong electrostatic and hydrogen-bonding inter­actions in the crystal packing.

## 3. Supra­molecular features

**Figure 2:**
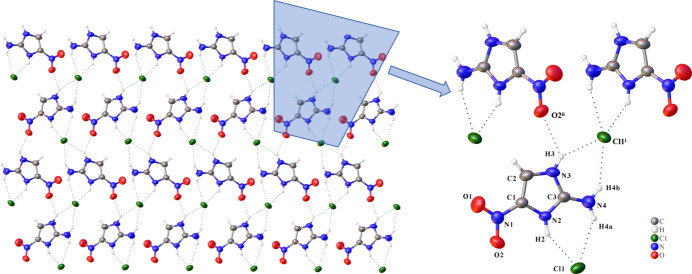
Hydrogen-bonding network of 2-amino-4-nitro­imidazole hydro­chloride [symmetry codes: (i) *x*, −*y* + 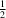, *z* + 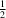; (ii) *x*, −*y* + 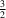, *z* − 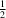].

The crystal structure of 2-amino-4-nitro­imidazole hydro­chloride features a sophisticated three-dimensional supra­molecular architecture constructed through an elaborate hydrogen-bonding network, as shown in Table 1 ▸ and Fig. 2 ▸. The chloride anion (Cl1) serves as a crucial structural bridge, participating in multiple N—H⋯Cl hydrogen bonds that connect the cations into an extensive three-dimensional framework. Within this network, Cl1 and adjacent cations form a characteristic 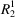
(6) hydrogen-bonding motif through N—H⋯Cl inter­actions, which serves as a key building block supporting the entire architectural framework. This network represents a classic example of charge-assisted hydrogen bonding, where the complementary electrostatic properties of the imidazolium cation and chloride anion enhance both the strength and directionality of the inter­molecular inter­actions. More precisely, Cl1 functions as a multipoint acceptor, engaging in four distinct N—H⋯Cl hydrogen bonds with precise geometric parameters. Two inter­actions originate from the reference mol­ecule: N2—H2⋯Cl1 (H2⋯Cl1 = 2.33 Å) and N4—H4*a*⋯Cl1 (H4*a*⋯Cl1 = 2.54 Å). Two additional connections are established with symmetry-related mol­ecules: N3—H3⋯Cl1^i^ (H3⋯Cl1^i^ = 2.40 Å) and N4—H4b⋯Cl1^i^ (H4b⋯Cl1^i^ = 2.50 Å) [symmetry code: (i) *x*, −*y* + 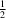, *z* + 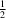]. The geometric consistency of these inter­actions, with *D*—H⋯*A* angles consistently exceeding 140°, confirms their strength and directional preference. An auxiliary N3—H3⋯O2^ii^ hydrogen bond [H3⋯O2^ii^ = 2.514 (2) Å; symmetry code: (ii) *x*, −*y* + 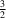, *z* + 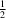] provides additional consolidation within the overall architecture. Although this inter­action is weaker, as indicated by the suboptimal angle, it serves as an important structural element that cross-links the primary hydrogen-bonded framework, adding dimensionality and robustness to the supra­molecular assembly.

The hydrogen-bonding network propagates in three dimensions with distinct directional preferences. Well-defined chains extend along the *b*-axis direction through the N—H⋯Cl hydrogen bonds, creating linear motifs that serve as the primary structural elements. These chains are subsequently inter­connected in the *ac* plane through a combination of N—H⋯Cl and N—H⋯O inter­actions, establishing layered substructures within the crystal. This hierarchical organization – from one-dimensional chains to two-dimensional layers and finally to a three-dimensional network – demonstrates the sophisticated level of structural control achievable through charge-assisted hydrogen bonding.

**Figure 3:**
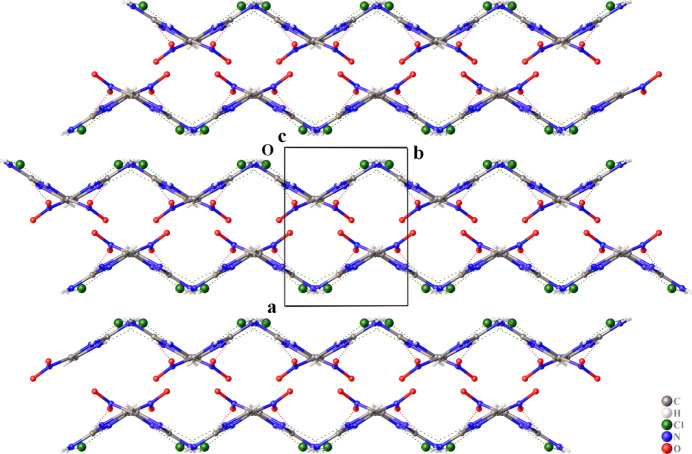
View of the crystal packing in the structure of 2-amino-4-nitro­imidazole hydro­chloride viewed along the *c* axis.

Beyond these specific directional inter­actions, the three-dimensional framework is further consolidated by van der Waals forces operating between adjacent mol­ecular layers (Fig. 3 ▸). These ubiquitous though weaker inter­actions play a crucial role in filling the voids within the crystal structure and providing additional cohesive energy between the hydrogen-bonded layers. The van der Waals contacts ensure efficient space filling and contribute to the overall lattice energy, while the directional hydrogen bonds define the specific mol­ecular arrangement.

The inter­play between the strong, directional charge-assisted hydrogen bonds and the omnipresent, non-directional van der Waals contacts creates a robust and highly stable supra­molecular framework. This structural pattern is consistent with observations in other imidazolium chloride salts (Liao *et al.*, 2011 ▸), highlighting the general importance of charge-assisted hydrogen bonding in ionic crystal engineering.

## 4. Hirshfeld surface analysis

**Figure 4:**
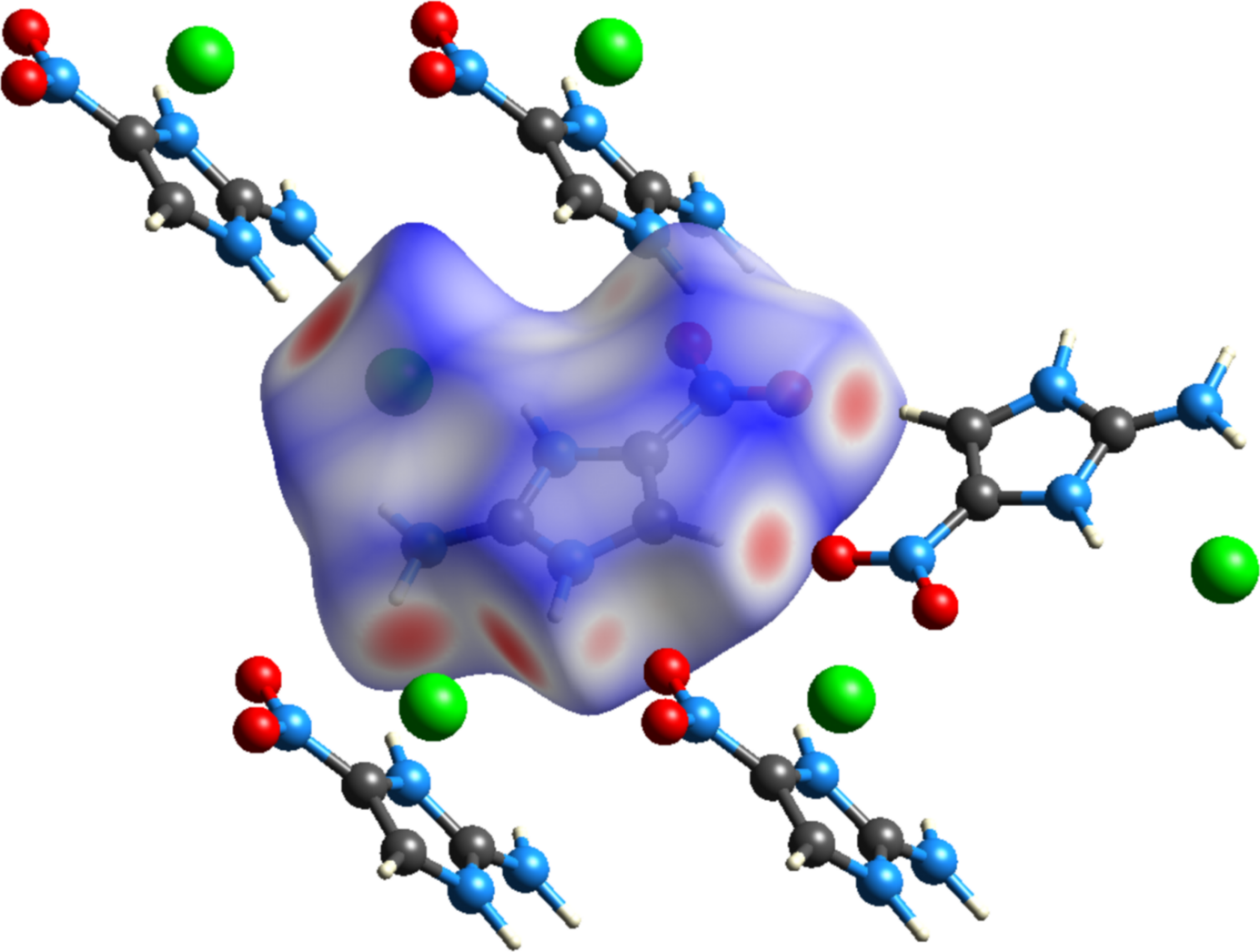
View of the three-dimensional Hirshfeld surface of 2-amino-4-nitro­imidazole hydro­chloride.

Hirshfeld surface analysis was employed to qu­anti­tatively investigate the inter­molecular inter­actions in 2-amino-4-nitro­imidazole hydro­chloride. The three-dimensional Hirshfeld surface mapped over normalized contact distance provides clear visualization of the inter­molecular contacts, as shown in Fig. 4 ▸. The surface exhibits distinct red spots in regions corresponding to close contacts, particularly near the chloride anion (Cl1), nitro group oxygen atoms (O1, O2), and hydrogen atoms of both the protonated imidazolium N3—H and amino N4—H groups. These characteristic red regions unequivocally identify the key participants in the hydrogen-bonding network.

**Figure 5:**
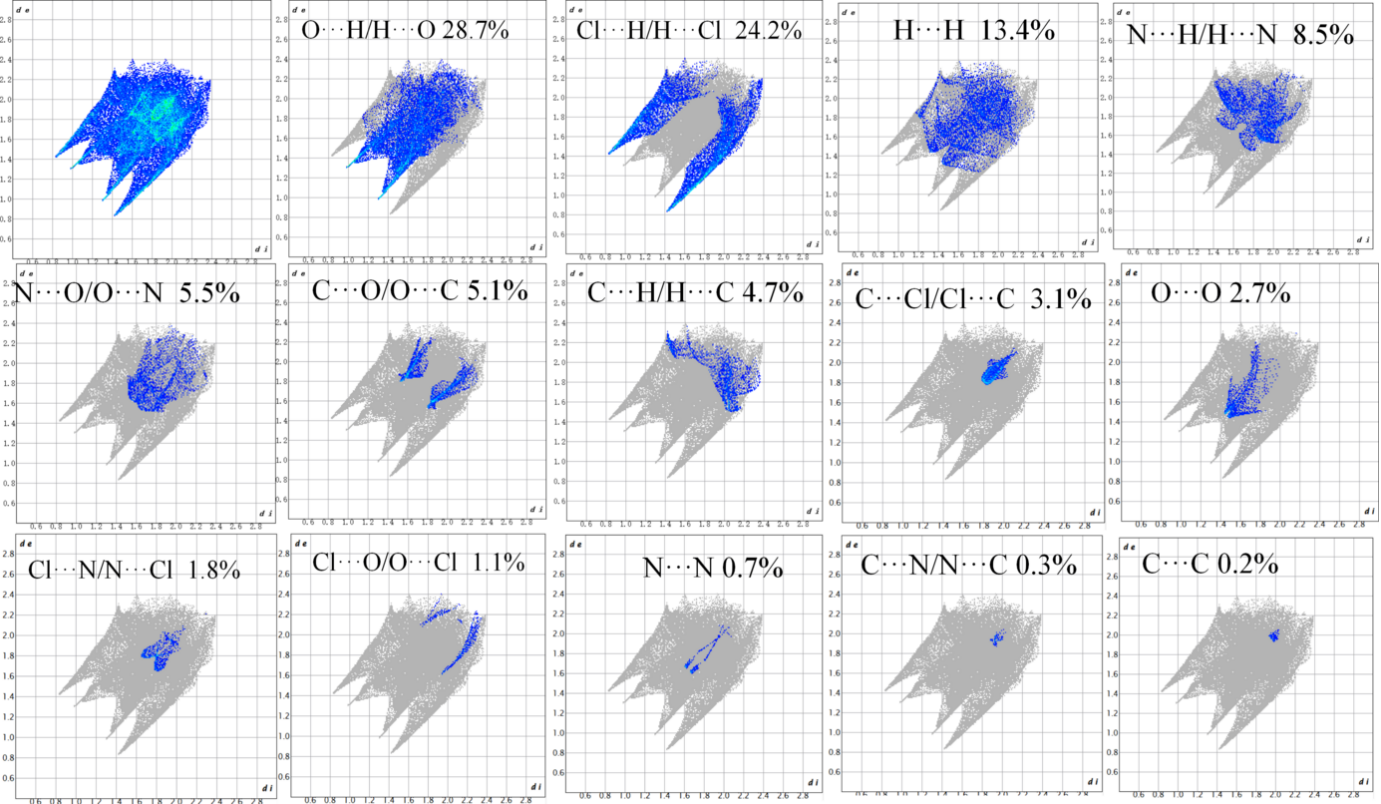
The two-dimensional fingerprint plots for 2-amino-4-nitro­imidazole hydro­chloride, showing all inter­actions and different contact types. The *d*_i_ and *d*_e_ values represent the closest inter­nal and external distances (in Å) from given points.

The qu­anti­tative decomposition of the Hirshfeld surface reveals several significant inter­action types (Fig. 5 ▸). O⋯H/H⋯O contacts represent the most substantial contribution at 28.7%, primarily attributed to N—H⋯O and C—H⋯O hydrogen bonds involving the nitro group oxygen atoms as acceptors. Particularly noteworthy is the considerable contribution from C⋯O/O⋯C contacts (5.1%), which provides clear evidence for the presence of C—H⋯O hydrogen bonds in the crystal structure. This type of weak hydrogen bonding, though less energetically favorable than conventional N—H⋯O bonds, plays a structurally significant role in the overall crystal packing.

Cl⋯H/H⋯Cl inter­actions constitute the second largest contribution at 24.2%, reflecting the formation of strong N—H⋯Cl hydrogen bonds between the chloride anion and both the imidazolium and amino N—H donors. This pattern is characteristic of hydro­chloride salts and has been consistently observed in related nitro­gen-rich heterocyclic compounds (Belfilali *et al.*, 2015 ▸). The coexistence of these directional N—H⋯Cl hydrogen bonds creates a robust ionic framework that significantly influences the mol­ecular arrangement.

Additional contributions include H⋯H contacts (13.4%), indicative of van der Waals inter­actions that contribute to efficient mol­ecular close-packing, and N⋯H/H⋯N inter­actions (8.5%), representing N—H⋯N hydrogen bonds within the structure. The minimal contributions from C⋯C (0.2%) and C⋯N/N⋯C (0.3%) contacts suggest that conventional π–π stacking inter­actions play a negligible role in the crystal packing, which is typical for polar ionic compounds where strong, directional hydrogen bonds dominate the supra­molecular architecture. The two-dimensional fingerprint plots provide further insight into the nature of these inter­actions. The plot for O⋯H/H⋯O contacts displays characteristic symmetric spikes, consistent with well-defined hydrogen-bonding geometry. Similarly, the Cl⋯H/H⋯Cl fingerprint shows sharp, distinctive spikes, reflecting the strong ionic hydrogen-bonding environment around the chloride anion.

In conclusion, the crystal packing of 2-amino-4-nitro­imidazole hydro­chloride is predominantly consolidated by a sophisticated hierarchy of inter­molecular inter­actions. Strong N—H⋯Cl and N—H⋯O hydrogen bonds form the primary structural framework, while weaker C—H⋯O hydrogen bonds and van der Waals inter­actions provide additional consolidation, collectively generating a cohesive three-dimensional supra­molecular architecture. This complementary inter­play of strong and weak forces follows established patterns observed in pharmaceutical salts and energetic materials, where such combinations frequently enhance structural stability and influence material properties (Richter *et al.*, 2020 ▸).

## 5. Synthesis and crystallization

A suspension of 2,4-di­nitro­imidazole (0.32 g, 2.0 mmol) in glacial acetic acid (25 mL) was treated with iron powder (0.37 g, 6.6 mmol) at room temperature. The reaction mixture was stirred for 30 minutes, then filtered through a sintered glass funnel. The filtrate was carefully poured into ice-cold water, and the pH was adjusted to 4–6 using dilute sodium hydroxide solution. The aqueous mixture was extracted with ethyl acetate, and the combined organic layers were dried over anhydrous sodium sulfate. After filtration, the solvent was removed under reduced pressure to yield the crude product as a brown solid.

Initial attempts to grow single crystals of the free base (2-amino-4-nitro­imidazole) from various solvent systems were unsuccessful. However, acidification with 10% hydro­chloric acid followed by slow evaporation yielded high-quality single crystals of the hydro­chloride salt suitable for X-ray diffraction analysis. The target compound, 2-amino-4-nitro­imidazole hydro­chloride, was ultimately obtained in pure form through recrystallization from 10% HCl solution.

## 6. Refinement details

Crystal data, data collection and structure refinement details are summarized in Table 2 ▸. Hydrogen atoms were placed in calculated positions and refined using a riding model: C—H = 0.95 Å, N—H = 0.88 Å with *U*_iso_(H) = 1.2*U*_eq_ of the parent atom.

## Supplementary Material

Crystal structure: contains datablock(s) global, I. DOI: 10.1107/S2056989025011399/ee2023sup1.cif

Structure factors: contains datablock(s) I. DOI: 10.1107/S2056989025011399/ee2023Isup2.hkl

Supporting information file. DOI: 10.1107/S2056989025011399/ee2023Isup3.cml

CCDC reference: 2517048

Additional supporting information:  crystallographic information; 3D view; checkCIF report

## Figures and Tables

**Table 1:** Hydrogen-bond geometry (Å, °)

*D*—H⋯*A*	*D*—H	H⋯*A*	*D*⋯*A*	*D*—H⋯*A*
N2—H2⋯Cl1	0.86	2.33	3.0686 (13)	144
N3—H3⋯Cl1^i^	0.86	2.40	3.1198 (15)	142
N3—H3⋯O2^ii^	0.86	2.51	3.0804 (19)	124
N4—H4*a*⋯Cl1	0.86	2.54	3.2595 (16)	142
N4—H4*b*⋯Cl1^i^	0.86	2.50	3.2336 (16)	144

Symmetry codes: (i) 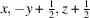; (ii) 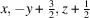.

**Table 2:** Experimental details

Crystal data	
Chemical formula	C_3_H_5_N_4_O_2_^+^·Cl^−^
*M*_r_	164.55
Crystal system, space group	Monoclinic, *P*2_1_/*c*
Temperature (K)	293
*a*, *b*, *c* (Å)	8.2117 (9), 6.3777 (6), 12.7216 (12)
β (°)	91.626 (3)
*V* (Å^3^)	665.98 (11)
*Z*	4
Radiation type	Mo *K*α
μ (mm^−1^)	0.52
Crystal size (mm)	0.13 × 0.12 × 0.09

Data collection	
Diffractometer	Bruker PHOTON II
Absorption correction	Multi-scan (*SADABS*; Krause *et al.*, 2015 ▸)
*T*_min_, *T*_max_	0.551, 0.746
No. of measured, independent and observed [*I* ≥ 2u(*I*)] reflections	5324, 1523, 1312
*R*_int_	0.035
(sin θ/λ)_max_ (Å^−1^)	0.652

Refinement	
*R*[*F*^2^ > 2σ(*F*^2^)], *wR*(*F*^2^), *S*	0.037, 0.096, 1.06
No. of reflections	1523
No. of parameters	91
H-atom treatment	All H-atom parameters refined
Δρ_max_, Δρ_min_ (e Å^−3^)	0.25, −0.29

Computer programs: *APEX5* (Bruker, 2023 ▸), *SAINT* (Bruker, 2019 ▸), *SHELXT2018/2* (Sheldrick, 2015 ▸), *OLEX2.refine* (Bourhis *et al.*, 2015 ▸) and *OLEX2* (Dolomanov *et al.*, 2009 ▸).

## supplementary crystallographic information

### 2-Amino-4-nitro-1H-imidazol-3-ium chloride Crystal data

**Table:**

C_3_H_5_N_4_O_2_^+^·Cl^−^	*F*(000) = 336.836
*M**_r_* = 164.55	*D*_x_ = 1.641 Mg m^−^^3^
Monoclinic, *P*2_1_/*c*	Mo *K*α radiation, λ = 0.71073 Å
*a* = 8.2117 (9) Å	Cell parameters from 2844 reflections
*b* = 6.3777 (6) Å	θ = 2.5–27.6°
*c* = 12.7216 (12) Å	µ = 0.52 mm^−^^1^
β = 91.626 (3)°	*T* = 293 K
*V* = 665.98 (11) Å^3^	Block, colourless
*Z* = 4	0.13 × 0.12 × 0.09 mm

### 2-Amino-4-nitro-1H-imidazol-3-ium chloride Data collection

**Table:**

Bruker PHOTON II diffractometer	1523 independent reflections
Radiation source: Bruker D8 VENTURE TXS PHOTON II	1312 reflections with *I*≥ 2u(*I*)
Detector resolution: 7.41 pixels mm^-1^	*R*_int_ = 0.035
ω and φ shutterless scans	θ_max_ = 27.6°, θ_min_ = 2.5°
Absorption correction: multi-scan (SADABS; Krause *et al.*, 2015)	*h* = −10→10
*T*_min_ = 0.551, *T*_max_ = 0.746	*k* = −8→8
5324 measured reflections	*l* = −15→16

### 2-Amino-4-nitro-1H-imidazol-3-ium chloride Refinement

**Table:**

Refinement on *F*^2^	0 restraints
Least-squares matrix: full	All H-atom parameters refined
*R*[*F*^2^ > 2σ(*F*^2^)] = 0.037	*w* = 1/[s^2^(*F*_o_^2^) + (0.0388*P*)^2^ + 0.2321*P*] where *P* = (*F*_o_^2^ + 2*F*_c_^2^)/3
*wR*(*F*^2^) = 0.096	(Δ/σ)_max_ = 0.0003
*S* = 1.06	Δρ_max_ = 0.25 e Å^−^^3^
1523 reflections	Δρ_min_ = −0.29 e Å^−^^3^
91 parameters	

### 2-Amino-4-nitro-1H-imidazol-3-ium chloride Special details

**Table:**

Refinement. The final refinement converged to R_1=0.0370 for 1312 reflections with I>2σ(I) and ωR_2_=0.0955 for all 1523 independent reflections. The goodness-of-fit on F^2^ was 1.057. In the final difference Fourier map, the maximum and minimum residual electron density peaks were 0.249 and -0.290 eÅ^-3^, respectively.

### 2-Amino-4-nitro-1H-imidazol-3-ium chloride Fractional atomic coordinates and isotropic or equivalent isotropic displacement parameters (Å^2^)

**Table:**

	*x*	*y*	*z*	*U*_iso_*/*U*_eq_	
Cl1	0.89095 (6)	0.35044 (7)	0.12243 (3)	0.05441 (18)	
N2	0.75517 (17)	0.5736 (2)	0.31487 (9)	0.0386 (3)	
H2	0.77073 (17)	0.5608 (2)	0.24860 (9)	0.0463 (4)*	
O2	0.6497 (2)	0.9190 (2)	0.21110 (10)	0.0613 (4)	
N3	0.7533 (2)	0.5142 (2)	0.48269 (10)	0.0471 (4)	
H3	0.7686 (2)	0.4540 (2)	0.54264 (10)	0.0565 (4)*	
O1	0.5401 (2)	1.0442 (2)	0.35036 (13)	0.0713 (5)	
N1	0.61748 (19)	0.9106 (2)	0.30400 (12)	0.0466 (4)	
N4	0.8887 (2)	0.2681 (2)	0.37526 (12)	0.0580 (4)	
H4a	0.9168 (2)	0.2338 (2)	0.31297 (12)	0.0696 (5)*	
H4b	0.9158 (2)	0.1898 (2)	0.42795 (12)	0.0696 (5)*	
C1	0.6751 (2)	0.7355 (3)	0.36250 (12)	0.0387 (3)	
C3	0.8045 (2)	0.4396 (3)	0.38987 (12)	0.0405 (4)	
C2	0.6737 (2)	0.7002 (3)	0.46675 (13)	0.0466 (4)	
H2a	0.6280 (2)	0.7850 (3)	0.51754 (13)	0.0559 (5)*	

### 2-Amino-4-nitro-1H-imidazol-3-ium chloride Atomic displacement parameters (Å^2^)

**Table:**

	*U*^11^	*U*^22^	*U*^33^	*U*^12^	*U*^13^	*U*^23^
Cl1	0.0764 (4)	0.0530 (3)	0.0343 (2)	−0.0076 (2)	0.0095 (2)	−0.01106 (17)
N2	0.0507 (8)	0.0421 (7)	0.0230 (6)	0.0027 (6)	−0.0004 (5)	−0.0010 (5)
O2	0.0884 (11)	0.0546 (8)	0.0406 (7)	−0.0041 (7)	−0.0022 (7)	0.0113 (6)
N3	0.0636 (9)	0.0533 (8)	0.0243 (6)	0.0047 (7)	0.0003 (6)	0.0031 (6)
O1	0.0810 (11)	0.0628 (9)	0.0699 (10)	0.0293 (8)	−0.0026 (8)	−0.0072 (7)
N1	0.0536 (9)	0.0427 (8)	0.0430 (8)	0.0008 (6)	−0.0070 (6)	0.0004 (6)
N4	0.0813 (12)	0.0525 (9)	0.0401 (8)	0.0200 (8)	−0.0002 (8)	0.0024 (7)
C1	0.0444 (8)	0.0418 (8)	0.0299 (7)	0.0005 (7)	0.0003 (6)	−0.0017 (6)
C3	0.0489 (9)	0.0434 (8)	0.0291 (7)	0.0011 (7)	−0.0013 (6)	−0.0004 (6)
C2	0.0555 (10)	0.0535 (10)	0.0308 (7)	0.0059 (8)	0.0038 (7)	−0.0051 (7)

### 2-Amino-4-nitro-1H-imidazol-3-ium chloride Geometric parameters (Å, º)

**Table:**

N2—H2	0.8600	O1—N1	1.224 (2)
N2—C1	1.374 (2)	N1—C1	1.416 (2)
N2—C3	1.335 (2)	N4—H4a	0.8600
O2—N1	1.220 (2)	N4—H4b	0.8600
N3—H3	0.8600	N4—C3	1.310 (2)
N3—C3	1.351 (2)	C1—C2	1.345 (2)
N3—C2	1.366 (2)	C2—H2a	0.9300
			
C1—N2—H2	126.12 (8)	C3—N4—H4b	120.0
C3—N2—H2	126.12 (9)	N1—C1—N2	121.18 (14)
C3—N2—C1	107.76 (13)	C2—C1—N2	109.04 (14)
C3—N3—H3	125.21 (9)	C2—C1—N1	129.66 (15)
C2—N3—H3	125.21 (9)	N3—C3—N2	107.67 (14)
C2—N3—C3	109.59 (13)	N4—C3—N2	125.70 (15)
O1—N1—O2	124.47 (16)	N4—C3—N3	126.62 (15)
C1—N1—O2	117.75 (15)	C1—C2—N3	105.92 (14)
C1—N1—O1	117.77 (15)	H2a—C2—N3	127.04 (9)
H4b—N4—H4a	120.0	H2a—C2—C1	127.04 (10)
C3—N4—H4a	120.0		
			
N2—C1—N1—O2	−4.27 (19)	O1—N1—C1—C2	−8.1 (2)
N2—C1—N1—O1	176.41 (17)	N1—C1—N2—C3	175.77 (16)
N2—C1—C2—N3	−0.22 (15)	N4—C3—N2—C1	−178.22 (19)
N2—C3—N3—C2	−1.35 (17)	N4—C3—N3—C2	178.0 (2)
O2—N1—C1—C2	171.27 (16)	C1—C2—N3—C3	0.96 (18)
N3—C3—N2—C1	1.18 (16)	C3—N2—C1—C2	−0.60 (15)
N3—C2—C1—N1	−176.18 (12)		

### 2-Amino-4-nitro-1H-imidazol-3-ium chloride Hydrogen-bond geometry (Å, º)

**Table:**

*D*—H···*A*	*D*—H	H···*A*	*D*···*A*	*D*—H···*A*
N2—H2···Cl1	0.86	2.33	3.0686 (13)	144
N3—H3···Cl1^i^	0.86	2.40	3.1198 (15)	142
N3—H3···O2^ii^	0.86	2.51	3.0804 (19)	124
N4—H4*a*···Cl1	0.86	2.54	3.2595 (16)	142
N4—H4*b*···Cl1^i^	0.86	2.50	3.2336 (16)	144

Symmetry codes: (i) *x*, −*y*+1/2, *z*+1/2; (ii) *x*, −*y*+3/2, *z*+1/2.
